# Viability of Cholera Virbio in Water of N. Y. Bay

**Published:** 1916-04

**Authors:** 


**Viability of Cholera Virbio in Water of N. Y. Bay.** A. J. Gelaire, Rosebank, N. Y., Med. Rec., Feb. 5. From culture experiments with water taken from the Bay, the following conclusions are reached: Multiplication does not occur but the vibrios survive from 7 to 47 days.

